# Legal Notes for Institutional Workers

**Published:** 1912-11-23

**Authors:** 


### LEGAL NOTES FOR INSTITUTIONAL WORKERS.

(The Editor will be pleased to answer Legal queries in this column.)

### Claim for Compensation by a Nurse.

In a recent case at Hanley it was alleged by the plaintiff, a nurse, that she contracted blood poisoning in her right hand while engaged attending respondent's late wife during confinement. She was engaged in November 1911 at a fixed fee of six guineas for a month. She began her duties on January 8; on the 10th of that month she developed signs of poisoning in the third finger of her right hand. Up to that time she was perfectly well; and, although there might have been some scratch on her finger, she had no knowledge of anything wrong. She had not been exposed to infection as she had been taking a holiday immediately before attending the patient. The finger became gradually worse and eventually it became permanently bent. In defence it was alleged (*a*) that a nurse was not a workman within the meaning of the Act; (*b*) that the injury was a disease, which did not arise from the accident; and (*c*) that the employment was of a casual nature to which the Act did not apply.

In giving judgment his Honour said everybody would be sorry for the nurse who had brought the proceedings. Claims founded on contracted disease were difficult to sustain, and only one or two had ever succeeded, because disease, in the ordinary acceptance of the term, did not come under the Act. It was said that this lady was not a workman under the Act at all, and he was inclined to think that argument was right. This was not a contract of service, but a contract for services at a fixed fee. On the second ground, he was not satisfied that this was not casual employment. Assuming the Act otherwise applied, the question was, Did the nurse in fact get the disease from which she was suffering by infection from the patient she was nursing? On the evidence he had heard all he could say was that he was not satisfied she did get the disease from the patient. Therefore, the claim failed.

### Unqualified Dentists.

Members of the hospital staff frequently come across patients who have been in the hands of the unregistered dentist. This kind of irregular practitioner, in order to conceal the fact that he is not qualified, calls himself a "teeth specialist," or adopts some other high-sounding title. It is important to notice that the *practice* of dentistry is not prohibited by law, but "no person may take or use the title of dentist or of dental practitioner, or any name, title, or addition implying that he is registered . . . or that he is a person specially qualified to practise dentistry unless he is registered." From the case of Robertson *v.* Hawkins, which was reported in the *Times* of November 1, it would seem that there is a certain method of holding himself out as registered which may in future bring the unregistered dentist within reach of the law. The London County Council required a school teacher to produce a dental certificate. The respondent, an unregistered practitioner, saw the teacher and extracted certain teeth. The teacher then handed him a form of certificate to fill up, saying he wanted it filled up by a registered dentist. The "dentist" filled up the form, telling the patient that he had given hundreds of such certificates. The Court (Lord Alverstone, Mr. Justice Channell, and Mr. Justice Avory) came to the conclusion that he had committed an offence within the meaning of the Act, laying stress on the fact that he had made a statement as to having given other certificates. While it is doubtful from this case whether the mere grant of one such certificate would constitute an offence, the decision will undoubtedly put a serious check on irregular practice. It only remains for the County Council and similar bodies to refuse to accept any but the certificate of a registered practitioner.

### A Doctor's Successful Claim for Fees.

In the King's Bench Division, before the Lord Chief Justice and a special jury, judgment was given in a claim by Dr. Benjamin Duke, of Clapham Common, for the recovery of £600 14s., against the executors of the late Edward Henry Brown, of Roehampton, for fees for medical attendance. The defendants admitted the attendances, but said that the charges were exorbitant, alleging also unnecessary visits. Sir James Goodhart gave evidence to the effect that daily attendance was in his opinion necessary. Sir James said he saw Mr. Brown in January 1911, when he was suffering from a failing heart, mouth trouble, and general bloodlessness. Mr. Cecil R. C. Lister, in charge of the electrical department of Middlesex Hospital, said he saw Mr. Brown in May 1910; he was suffering from carcinoma. He gave him radium treatment, and it was a question of daily or nearly daily attendance. The Lord Chief Justice said that the charge that the plaintiff had attended unnecessarily in order to make money was a serious one to make against a medical man. It was remarkable that the defendants had been unable to bring forward medical evidence to support their case. The jury returned a verdict for the plaintiff for the amount claimed.

### The Law on Fees.

There is, of course, no legal limit to the charges which a doctor may make for his attendance. That is a purely business matter between himself and his patient. When disputes arise each case is adjudged upon its own intrinsic merits; but it may be noted that patients (or executors) always experience great difficulty in proving their case. Mr. Brown's executors held that one guinea was an exorbitant fee for a general practitioner in a suburb to charge for each visit, but this contention was easily rebutted by the complainant, who was able to show that the patient lived five miles away, had always paid without complaint accounts compiled upon the same basis, and requested the doctor to continue in attendance after his relatives had expressed their feelings on the subject of the fees.

